# To sit or stand? A preliminary, cross sectional study to investigate if there is a difference in glenohumeral subluxation in sitting or standing in people following stroke

**DOI:** 10.1186/s40945-015-0006-9

**Published:** 2015-08-11

**Authors:** Nichola J. Hatton, Rachel C. Stockley

**Affiliations:** 1 Tameside General Hospital, Ashton-u-Lyne, Greater Manchester, OL6 9RW UK; 2 Department of Health Professions, Birley Campus, Manchester Metropolitan University, Manchester, M15 6GX UK

**Keywords:** Stroke, Subluxation, Glenohumeral, Postural control, Rehabilitation

## Abstract

**Background:**

Glenohumeral subluxation (GHS) is a common symptom following stroke. Many therapists postulate that GHS may be reduced if the base of support (BOS) is reduced and the centre of mass (COM) is raised as this requires greater postural muscle activity. However, there is little empirical evidence to support this practice.

**Objective:**

The aim of this preliminary study was to investigate if the amount of GHS alters from sitting to standing.

**Study design:**

A cross sectional, within-subject design in a convenience sample of 15 stroke patients with GHS was utilised.

**Methods:**

A prospective design was used with a single blinded tester who assessed GHS using the calliper method in sitting, standing and on return to sitting. Friedman and post hoc Wilcoxon tests showed that GHS was significantly reduced in standing compared to sitting (*p* <0.05) but this reduction was not maintained on return to sitting (*p* = 0.25).

**Conclusions:**

The results of this study are limited by its small size. However, these results indicate that reducing BOS during rehabilitation may improve GHS after stroke. Whilst the maintenance of benefit is not established, these findings suggest that reducing BOS as part of treatment may help patients with GHS. Further research is now required to replicate these results in a larger sample and to directly examine shoulder muscle activity to investigate which muscles may influence GHS in response to changing BOS. Future work could also aim to determine whether the reduction in GHS was directly attributable to a reduced BOS or the effort associated with moving from sitting to standing.

## Background

Recovery of the upper limb has been described as ‘notoriously poor’ following stroke [1] (p1). Studies have shown that up to 75 % of individuals with upper limb deficits have ongoing symptoms preventing normal activities of daily living at six months post stroke [2]. Whilst weakness within any muscle group will affect recovery, it has been noted that glenohumeral subluxation (GHS) in particular, will reduce the rehabilitation potential of the whole arm [1] and has been significantly associated with poor functioning of the upper limb [3].

The stability of the shoulder joint is dependent upon an active system comprising the contractile tissues of the rotator cuff muscles and larger muscles such as deltoid, biceps brachii and pectoralis major [4]. Collectively the rotator cuff muscles pull the humeral head into the glenoid cavity to stabilise and centralise it [5]. However, this function requires an intact neuromuscular- system [6] which may be compromised after stroke due to alterations in descending neural excitation.

Inferior GHS is common after stroke and is estimated to affect between 17 and 81 % of all patients after stroke [7]. For the purposes of this study, we described inferior GHS to be an inferior glenohumeral joint displacement as a result of the gravitational pull of the humerus [8] which is observable and/or palpable, typically as a dip in the smooth contour under the acromion process. It can cause considerable pain and results in reduced upper limb function for many [6]. It is recognised that alignment of the GH joint can alter in both the flaccid and spastic stages of paralysis following stroke creating malalignment in a variety of directions however, this study was concerned only with inferior GHS as a consequence of gravity.

Current treatment of GHS includes the use of external supports such as strappings, slings and pillows, although there are no firm conclusions regarding their effectiveness [7, 8, 9]. The use of functional electrical stimulation has also been investigated but is currently only recommended as part of a clinical trial in the UK as its effectiveness and efficacy is not yet clear [10].

Many therapists postulate that a person positioned with a small supporting surface or base of support (BOS) and high centre of mass (COM) will require greater postural muscle activity than if they are in a position with a larger BOS and lower COM [11, 12]. Consequently, if the muscles that maintain normal glenohumeral alignment are considered to be tonic in function [13] then altering BOS and COM could influence GHS during therapy and could be a beneficial treatment strategy for people with GHS after stroke. Therefore, this small study aimed to gather preliminary data to explore whether changing the size of BOS influenced the amount of GHS in individuals with this symptom after stroke.

## Methods

This study was a prospective, within subject, cross sectional uncontrolled study to measure the magnitude of GHS in sitting and standing. Participants were recruited from the local stroke population of a hospital in the North West of England, were above the age of 18, and met the following criteria:

- First stroke- identified on CT scan (ischaemic or haemorrhagic)
- Visible or palpable GHS in the affected shoulder in the sitting position (as identified by the individual’s usual therapist.
- A score of less than 5 errors (from 10) on the Short Portable Mental Status Questionnaire, to ensure the individual had both the ability to consent and follow test procedures [14].
- Ability to sit unassisted on a plinth with feet on the floor and be able to stand with minimal assistance, sustaining an upright posture for several minutes (with standby supervision if required). This was determined by the individual being capable of completing the first four items on the Berg Balance Scale [15].

Participants were excluded if they had a diagnosis of dementia or other neurological deficits which may interfere with shoulder stability or speech problems which impaired their ability to understand instructions or give consent. Additionally, individuals with shoulder pain in the affected arm at rest, with the arm hanging dependent, or those unable to participate in usual physiotherapy because of shoulder pain were not included.

Participants completed the Barthel Index and details of the time since stroke, type and side of stroke, age and gender were recorded. Participants were seated at the end of a variable height plinth so that the affected arm could be lengthened at the side of the body without obstruction. The participants sat with their hips and knees at 90 degrees and their feet flat on the floor. The thighs were fully supported on the plinth and the posterior point of contact marked with tape to ensure the same position on returning to sitting. The amount of GHS was measured using the calliper technique which has been shown to be reliable and valid in stroke patients [13] by a physiotherapy technical instructor (BR) trained in its use but who was blind to the aim of the study. She marked the tip of the acromion process with a non-permanent pen and then located the superior aspect of the head of humerus using palpation. The distance between the two points was established using a standard school calliper. This was then placed on a standard ruler and the distance noted in millimetres.

All participants were measured in sitting, then asked to stand. The amount of GHS was re-measured in standing immediately and, as soon as the measurements had been taken, participants were asked to sit. The amount of GHS was measured for a third time immediately upon the participant returning to the original sitting position. In each position (sitting, standing and return to sitting) the measurement was repeated three times and the average of the three values was used for analysis.

### Data analysis

To ascertain if there was a significant difference between the measures in each of the three positions (sitting, standing and return to sitting), a non-parametric repeated measures test (Friedman’s test) was used. Post-hoc Wilcoxon tests were used to establish differences between GHS measurements in sitting and standing. A *p* value of <0.05 was used for all tests. All data were analysed using SPSS® version 17.

Ethical approval was obtained from the Manchester Metropolitan University Faculty of Health and Social Care and National and Local Research Ethics committees. The trial was reported according to STROBE guidelines.

## Results

15 participants (6 males: mean age 61, range 46–83, SD 13 years) were recruited at a mean of 44 weeks following stroke (range 1–308: SD 79 weeks). Thirteen participants had been diagnosed with an ischaemic stroke, 8 had right sided weakness and 9 were out-patients.

Table 1 presents the participants’ characteristics.

**Table 1:** Participant characteristics

Participant No.	Gender	Age	Affected side	Type of stroke	Time since stroke (Weeks)	Barthel Index score (/20)
1	F	46	R	Parietal lobe infarct	308	20
2	M	66	L	MCA infarct	105	19
3	M	69	L	Thalamic infarct	48	14
4	M	53	R	Parietal/internal capsule infarct	11	16
5	F	46	R	Basal ganglia infarct	75	15
6	F	53	L	Frontoparietal infarct	17	17
7	M	48	L	MCA infarct	1	11
8	F	48	R	Haemorrhage	18	11
9	F	69	L	MCA infarct	16	13
10	F	58	R	Basal ganglia infarct	25	14
11	M	82	L	MCA infarct	3	10
12	F	50	L	MCA infarct	3	8
13	F	66	R	Parietal infarct	29	14
14	F	83	R	Basal ganglia infarct	1	9
15	M	80	R	MCA infarct	2	9

*R* right, *L* left, *MCA* middle cerebral artery

The Friedman test indicated that there was a significant difference in the amount of GHS between the three conditions (sitting, standing and return to sitting; *p* = 0.009). Wilcoxon tests showed that there was a significant reduction in GHS in standing compared to sitting (*p* = 0.009), with a significant increase in GHS when returning to sitting from standing (*p* = 0.017) as displayed in Table 2.

**Table 2:** Glenohumeral subluxation (GHS) in sitting, standing and return to sitting

Position	GHS (mm)	Range (mm)	95 % CI of the change in GHS in different positions			
			Initial sitting	*P*	Standing	*P*
Initial sitting	13	12 (8-21)	-	-		
Standing	9	16 (0-16)	−1.29 to -6.98	0.009	-	-
Return to sitting	12	17 (4-21)	−0.91 to 3.8	0.25	5.36 to 0.24	0.017

A negative value indicates a reduction in GHS. *CI* confidence interval

**Fig. 1:**
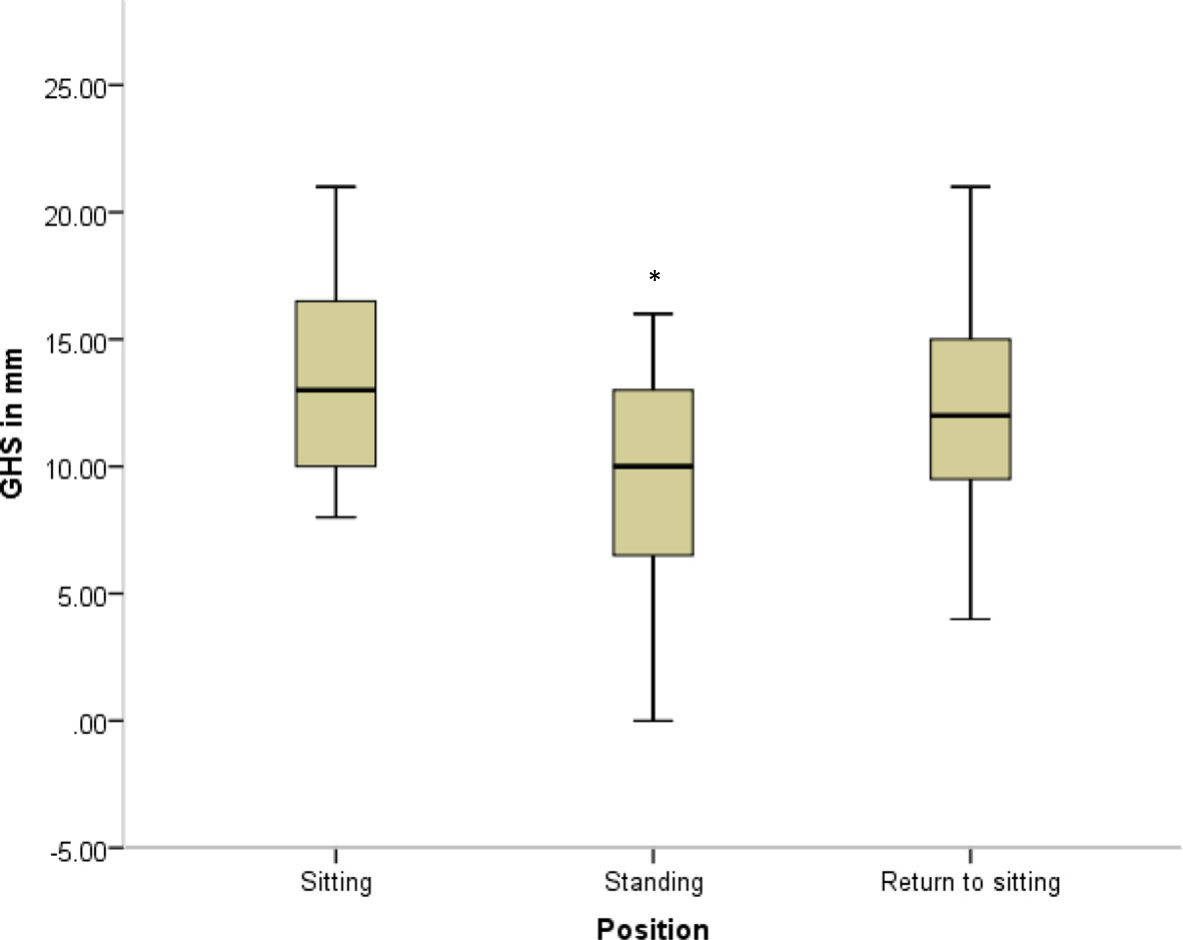
Boxplot to show measurements of glenohumeral subluxation (GHS) in sitting, standing and upon return to sitting. Lines indicate median GHS in each position with the lower and upper margins of the box indicating the 25th and 75th centiles respectively. Error bars show the range. *denotes significant differences (*p* < 0.05) in GHS from both sitting and return to sitting

There was no significant difference in GHS between initial sitting and return to sitting (*p* = 0.25). Figure 1 shows the median values and interquartile ranges for GHS in each position.

## Discussion

These results show that there was a statistically significant reduction in GHS in standing compared to sitting, suggesting that a reduction in the BOS also reduced the amount of GHS in participants after stroke.

There were several limitations to this study, most notably the small sample size. Nonetheless, the results support the practice of considering BOS of the patient when treating individuals following stroke [12, 16], as in standing there appears to be a greater potential for muscle recruitment and thus reduced GHS. However, these findings require verification in a larger study before clinical recommendations can be developed and therapists should balance the safety implications when reducing an individual’s BOS against any possible benefits in muscle activity.

This small study was unable to demonstrate the cause of the reduced subluxation seen on standing, but it is likely that the changes observed are as a result of greater activity within the rotator cuff. These muscles, with some deltoid activity provide the main component of control at the GH joint [17-19, 20]. Indeed, paralysis of the supraspinatus muscle in particular has been suggested to be a predictor of a greater risk of GHS by some [21]. Marieb (2004) [15] describes the rotator cuff tendons as being kept taut by the resting tone within the muscle and Edwards (2002) [5] suggests that local stabilisers, comprising slow oxidative (tonic) fibres, are largely fatigue resistant with long lasting but weak contractions. The findings of the current study lend some support to the notion that the rotator cuff muscles appear to act as postural muscles. The results also suggest that they increase their activity on standing and thereby reduce GHS.

The effect of postural control on GHS has not been explored within the literature. The mechanical factors relating to aetiology and treatment have been outlined but few studies have considered the effect of changing the base of support upon muscle activity and GHS and so we can only hypothesise the mechanisms by which these changes have occurred [9]. It is possible that the activation of the rotator cuff and subsequent reduction in GHS within the standing position may occur because different neurological pathways are activated, for example, the medial reticulospinal tract [18, 19]. In comparison to sitting, standing elicits greater sensory input from the lower limbs, particularly the soles of the feet which can increase extensor muscle activity via synergistic muscle patterns [15]. This process is likely to utilise propriospinal neurons which are located within the spinal cord. The longest of axons of these nerve cells lie within the medial pathways and can innervate both proximal and axial muscles creating co-activation, even in the presence of a damaged central nervous system [22].

Another potential contributor to a reduction in GHS on standing is that some tracts which can influence muscle activity at the shoulder may originate on the ipsilateral side of the brain and so would maintain innervations to the affected side after stroke. Such tracts include the medial reticulospinal pathway which creates activity within the postural muscles and limb extensors and the lateral vestibulospinal tract which is activated by changes in response to gravity [18, 19].

An alternative explanation of why GHS may reduce as BOS and COM rises could be that it is purely the effect of the effort required to overcome the inertia of sitting to achieve standing. During movements that require effort it is normal to have a generalised increase in activity elsewhere in the body [11]. It is therefore a limitation of this study that the reason for the reduction in GHS cannot be determined; this could be further examined in future work by asking participants to stand for longer to determine if an initial increase in muscular activity reduces.

Further research could also seek to identify if muscular activity is related to shoulder pain in GHS [22] and to see which muscles increase their activity on standing, to determine the mechanism by which GHS was observed to decrease [21]. It is now also necessary to evaluate if treatment with a smaller BOS has lasting benefits to upper limb function in individuals who have GHS after a stroke.

## Conclusions

Despite its limitations, the finding of the current study suggests that therapists should consider reducing a patient’s BOS, where is safe to do so, during treatments in order to activate postural muscles and in turn reduce GHS. The use of internally generated mechanisms to reduce GHS in this way, rather than passive supports such as slings or cuffs, may promote more function within the arm and provide a low cost and simple method to augment treatment.

Further research is now needed in a larger trial to verify these findings and to determine whether it is the effort associated with standing or the reduction in BOS which leads to GHS reduction. Future research could also investigate if GHS reduction can be sustained when standing for longer periods to increase the clinical relevance of these results.
